# Poverty, Tobacco, and Health: An Indian Scenario

**DOI:** 10.3329/jhpn.v27i3.3373

**Published:** 2009-06

**Authors:** J.P. Majra, A. Gur

**Affiliations:** ^1^ Department of Community Medicine, K.S. Hegde Medical Academy, Mangalore 575 018, India; ^2^ Department of Periodontics, A.B. Shetty Memorial Institute of Dental Sciences, Deralakatte, Mangalore 575 018, Karnataka, India

**Keywords:** Health, Poverty, Tobacco-use, India

## Abstract

Poverty and health have a two-way relationship. Poverty increases the vulnerability of people to disease, and sickness affects their income leading to poverty. Tobacco has been identified as a major avoidable cause of illness and premature death. In India, more than half of men and one-tenth of women use one or more forms of tobacco. Tobacco-use shows a clear and continual increase with decreasing wealth quintiles. Poor smokers, who are at a greater risk of illness, are also at a greater risk of not being treated or of falling into greater poverty if they seek treatment. Poor people spend money on tobacco that could be spent on food, shelter, education, and healthcare. These decisions can entrench families in an ongoing cycle of poverty and ill-health. The direct and indirect costs of tobacco-use are immense for national economy. This has positioned control of tobacco relevant in India's per suite to achieve the goals of poverty eradication and health for all.

## INTRODUCTION

Tobacco has been identified as a major avoidable cause of illness and premature death. Given the current patterns of consumption, it is estimated that tobacco will kill 80 million Indian males currently aged 0–34 year(s) (1). Apart from mortality, tobacco also accounts for a large portion of the burden of diseases of adults, which deeply affect families living in poverty.

## CURRENT TOBACCO-USE IN INDIA

In India, more than 57% of men and 11% of women use one or more forms of tobacco (2). The use of tobacco is more prevalent among both men and women in rural areas than in urban areas. In India, the use of tobacco shows a clear and continual increase with the decreasing wealth quintiles among both men and women (2). Seven in 10 men in the lowest wealth quintile consume tobacco products while four in 10 men in the highest wealth quintile do so. Twenty-two percent of women in the lowest wealth quintile consume tobacco. There is an equally clear and continual increase in tobacco-use with decreasing levels of education (2). Women and men from the scheduled castes and scheduled tribes (the poorest among poor in India) are more likely to use tobacco than those from other castes or tribes (2).

## TOBACCO COMPOUNDS THE PROBLEM OF POVERTY AND ILL-HEALTH

Anything that increases the likelihood that poor people will fall sick or be injured is especially problematic in low-income countries where healthcare, if accessible, is often very expensive, requiring significant private, under-the-table, and other payments. Poor smokers, who are at a greater risk of illness, are, therefore, also at a greater risk of not being treated or of falling into greater poverty if they seek treatment. In India, nearly 25% of people above the poverty-line, when admitted to a hospital, were below the line when discharged (3). Illness due to tobacco is not only caused by smoking or chewing. Those who harvest and cure tobacco frequently report poor health. Pesticides used in farming of tobacco also cause illnesses, including increased rates of depression and suicide among tobacco farmers (4).

## TOBACCO AFFECTS MATERNAL AND CHILD HEALTH

Women in many developing countries are being encouraged to take up smoking as a sign of increased equality. Women who use tobacco, in addition to sharing the same health risks as men, also experience difficulty in becoming pregnant, at an increased risk of pregnancy-related complications, premature birth, low-birthweight infants, stillbirths, and infant death. Tobacco also contributes to child morbidity through exposure to second-hand smoking. Second-hand smoking has been associated with infections in the lower respiratory tract, sudden infant death syndrome, and asthma in children (6). Research has shown that smoking of cigarettes may impact negatively on breastfeeding. Breastfeeding is nearly universal in India, and children, in particular among the poor, are nutritionally dependent on breastmilk. Volume of milk production is reduced among smoking mothers, and their milk contains less fat than the milk produced by non-smoking mothers (7). This reduction in the quality and quantity of breastmilk places them at a risk of malnutrition and infectious illnesses (8). The use of tobacco by pregnant and breastfeeding women in India is about equal to the use of tobacco by women who are neither pregnant nor breastfeeding, indicating that women who use tobacco may be unaware of the negative reproductive consequences of tobacco-use (2).

## OPPORTUNITY COST OF TOBACCO-USE AMONG THE POOREST

Several reasons have been offered for why smoking is more common among the poor. They may be less aware of the risks, or they may use nicotine as a self-medication for ailments they falsely believe that tobacco will relieve (9). They may also perceive tobacco as a ‘reward’, as one of the few pleasurable things that they can do for themselves (9). Perhaps, poor people may feel that they have less to lose from future illness because they see no future to look forward to or for which to plan. Another theory is that poor people become more physiologically addicted to nicotine as measured by nicotine metabolites (9).

For the poor, tobacco-use has a very high opportunity cost, in that it diverts spending from basic needs. A number of studies have indicated that poor people spend money on tobacco that could, in theory, be spent on other goods, such as food, shelter, education, and healthcare. Shah and Vaite reported that, of 400 street-children in Mumbai, most of whom earned less than US$ 2 a day, half smoked cigarettes or other locally-made tobacco products. They spent up to 21% of their income on tobacco products, far more than they spent on food, education, clothing, or savings (10).

At present, in India, 28% of children aged 6-17 years are not in school (2). Poverty and child labour are key reasons why parents do not send their children to school. Many industries in developing countries, including India, rely on child labour, including the tobacco industry, particularly tobacco cultivation or bidi-rolling (making hand-rolled cigarettes).

Apart from individuals and their families, the direct and indirect costs of tobacco-use are immense for national economies through its negative effects on health and productivity. In 2000, the Indian Council of Medical Research estimated the costs of three major tobacco-related diseases (cancer, heart diseases, and chronic obstructive lung disease) in India at US$ 5.8 billion, considerably in excess of the direct contribution of the tobacco industry to Indian government revenue of about $1.5 billion (11). Although these figures do not include lost productivity when workers become ill or prematurely die, they represent significant underestimates of total costs. India is the most populous low-income country with about one-fourth of her population living below the poverty-line. This has positioned control of tobacco relevant in India's per suite to achieve the goals of poverty eradication and health for all.

## WHAT CAN BE DONE?

Law can prove to be useful in curbing the tobacco epidemic to the extent it is implemented and enforced (12). Therefore, to curb the tobacco-use menace in the presence of motivating factors, such as social acceptance and positive attitude towards tobacco-use, a comprehensive approach is needed that also addresses the issues, such as misconceptions in the society regarding tobacco, creating a supportive environment by changing social norms, and medical support to treat nicotine dependence for those who wish to quit tobacco, in addition to the stringent legislation implemented in word and spirit.

## CONCLUSION

Research has repeatedly shown how important education is for better health. Educated parents are more likely to make appropriate decisions with respect to the health of their children (2). Education is also essential to enable individuals to lift themselves out of poverty. Figures for poor households that contain tobacco-users often show expenditure on tobacco at around 10% of all household expenditure. These decisions can entrench families in an ongoing cycle of poverty, as the very investments necessary to lift family members out of poverty are foregone in favour of an addictive drug.
